# Rainbow Trout IgM^+^ B Cells Preferentially Respond to Thymus-Independent Antigens but Are Activated by CD40L

**DOI:** 10.3389/fimmu.2019.02902

**Published:** 2019-12-17

**Authors:** Aitor G. Granja, Pedro Perdiguero, Alba Martín-Martín, Patricia Díaz-Rosales, Irene Soleto, Carolina Tafalla

**Affiliations:** Animal Health Research Center (CISA-INIA), Madrid, Spain

**Keywords:** teleost fish, B cells, IgM, extrafollicular responses, CD40L

## Abstract

In the absence of class switch recombination and germinal centers, the mechanisms through which B cells from teleost fish mount extrafollicular immunoglobulin M (IgM) memory responses remains mostly unexplored. In this report, we demonstrate that teleost IgM^+^ B cells respond to CD40L, a thymus-dependent activation signal, similarly to mammalian B2 cells. However, when stimulated with different types of antigens, fish IgM^+^ B cells only reach a general activation state in response to antigens cataloged as thymus-independent 1 (TI-1) in mammals, as established through both functional assays and RNA sequencing. Interestingly, fish IgM^+^ B cells remained completely unresponsive to TI-2 antigens, suggesting that the engagement of innate receptors provided by TI-1 antigens is required for the activation of teleost B cells. Finally, a synergy between CD40L and TI-1 antigens was also demonstrated, further supporting that there is no clear dichotomy between thymus-dependent and TI responses in teleost fish.

## Introduction

The classical dogma in mammalian immunology holds that B cell memory can only be achieved within germinal centers (GCs). For this, conventional B cells (B2 cells) are activated in response to thymus-dependent (TD) antigens within the lymphoid follicles, triggering the formation of GCs. Within these sites, the close interaction with T follicular helper cells stimulates B2 cells to divide and differentiate into antibody-secreting cells, reaching a terminal state of plasma cell (PC) or into memory B cells (1). Among the signals involved in T cell/B cell cooperation, the interaction between CD40 expressed on B cells and its ligand (CD40L) transitionally expressed on activated T cells, is of critical importance (2). In consequence, cross-linking of CD40 stimulates the formation of GCs and promotes B cell survival, proliferation and differentiation (2). Therefore, this TD pathway requires two signals, being “signal 1” that delivered through the B cell receptor (BCR) after recognition of the antigen, and “signal 2” the cognate help from T cells (mainly CD40 cross-linking) (3). Alternative to this TD response, mammals have additional thymus-independent (TI) pathways to elicit faster antibody responses that are mainly orchestrated by innate B cell subsets, mainly B1 cells or marginal zone (MZ) B cells. These TI responses do not require T cell cooperation but are activated by direct recognition of pathogen motifs or BCR stimulation (4). TI antigens have been classically classified as type 1 (TI-1) or type 2 (TI-2) depending on whether they can stimulate B cells in CBA/N mice, which have an X-linked immunological defect caused by deficiency in the kinase Btk (5). Thus, antigens that do not elicit responses in CBA/N mice are designated TI-2, while those that elicit antibody responses in the absence of Btk are cataloged as TI-1. TI-1 antigens provide a second signal via Toll-like receptors (TLRs) and include microbial products such as lipopeptides, lipopolysaccharide (LPS), microbial CpG DNA, viral RNA, or certain viral coat proteins (6). TI-2 antigens, on the other hand, are multivalent polysaccharides or large antigens with repetitive structures that can extensively cross-link the BCR (7). In this perspective, the established line of thought in mammalian immunology was that a long-term humoral immunological memory is established exclusively with T cell cooperation. However, in the light of recent investigations, it seems that mammals can acquire long-term immunological memory through alternative mechanisms that question this fixed dichotomy between TD and TI responses (7–12). Considering these studies, it now seems that there are multiples routes to achieve B cell memory in mammals (13) that include variations of the classically designated TD and TI responses.

The adaptive immune system arose approximately 500 million years ago in jawed fish. Thus, jawed fish (both cartilaginous and teleost fish) constitute the first animal group that expresses most of the essential elements needed to mount an adaptive immune response, while these elements are lacking in jawless vertebrates (14). In teleost fish, antibody responses are based on three immunoglobulin (Ig) isotypes, namely, IgM, IgD, and IgT, being the latter a fish-specific Ig (15) expressed on an independent B cell linage postulated as a dedicated mucosal cell type (16, 17). Although a role for IgT in systemic responses has also been reported in some studies (18–20), in teleost fish, in the absence of class switch recombination (CSR), systemic responses seem to rely exclusively on unswitched antibodies, predominantly IgM. On the other hand, teleost fish do not have lymph nodes or generate cognate GCs (21). These structural differences suggest that B cell responses in teleost could be assimilated to mammalian extrafollicular IgM responses. In this context, it becomes quite interesting to investigate how early vertebrates, such as rainbow trout (*Oncorhynchus mykiss*), respond to TI and TD antigens, or how different stimulatory signals synergize to activate B cells in these species. Thus, in this report, we demonstrate for the first time that fish IgM^+^ B cells respond to CD40L similarly to mammalian B2 cells, since incubation of splenocytes with recombinant rainbow trout CD40L upregulated the survival, proliferation, and promoted the differentiation of IgM^+^ B cells to PCs. Despite their capacity to respond to a cognate T cell signal such as CD40L, fish IgM^+^ B cells were only significantly activated by 2,4,6-trinitrophenyl hapten conjugated to LPS (TNP-LPS), an antigen cataloged as TI-1 in mammals, very faintly by a model TD antigen, while they remained completely unresponsive to TNP-Ficoll, an antigen classified as TI-2 in mammals. This differential response of fish B cells to TI-1 and TD antigens was further investigated through a complete transcriptomic analysis, which confirmed that although TD antigens promoted the regulation of specific immune pathways, an extensive cellular activation state was only achieved in response to a TI-1 antigen such as TNP-LPS. Finally, experiments in which CD40L was combined with TD and TI-1 revealed a synergy between CD40L and TI-1 antigens. Therefore, our results suggests that in teleost engagement of innate receptors such as TLRs is an essential step required for the activation of B cells. Furthermore, the fact that CD40L stimulation synergized with TI-1 responses further supports that there is not a clear dichotomy between TD and TI responses in teleost fish, whereas different degrees of activation are reached by B cells through the accumulation of different types of stimulatory signals.

## Materials and Methods

### Experimental Fish

Healthy specimens of female rainbow trout (*Oncorhynchus mykiss*) of ~50–70 g were obtained from Centro de Acuicultura El Molino (Madrid, Spain). Fish were maintained at the Animal Health Research Center laboratory at 16°C with a recirculating water system and 12:12 h light/dark photoperiod. Fish were fed twice a day with a commercial diet (Skretting, Spain). Before any experimental procedure, fish were acclimatized to laboratory conditions for 2 weeks, and during this period, no clinical signs were ever observed. The experiments described comply with the Guidelines of the European Union Council (2010/63/EU) and were previously approved by the Ethics committee from the Instituto Nacional de Investigación y Tecnología Agraria y Alimentaria (INIA; Code CEEA PROEX002/17).

### Reagents

Lipopolysaccharide (LPS) from *Escherichia coli* O26:B6, polyinosinic–polycytidylic acid (poly I:C), concanavalin A (ConA) from *Canavalia ensiformis*, collagenase type IV from *Clostridium histolyticum*, cytochalasin B from *Drechslera dematioidea* (cyto B), and benzocaine were purchased from Merck Millipore and used at concentrations previously described (22, 23). 2,4,6-Trinitrophenyl hapten conjugated to keyhole limpet hemocyanin (TNP-KLH), TNP-LPS, and TNP-Ficoll were acquired from LGC Biosearch Technologies. In all the experiments described in this study, these antigens were used at a concentration of 5 μg/ml, established after performing initial experiments with a wide dose range (data not shown).

### Leukocyte Isolation

Single-cell suspensions from spleen, skin, head kidney, gills, and intestine were prepared by pushing the tissues through 100 μm nylon cell strainers (BD Biosciences) with L-15 medium containing 100 IU/ml penicillin, 100 μg/ml streptomycin (P/S), 10 U/ml heparin, and 5% fetal calf serum (FCS) (Thermo Fischer Scientific). In the case of intestine and skin, before cell extraction, tissue samples were digested as previously described (23). All cell suspensions were then placed onto 30/51% Percoll (GE Healthcare) density gradients and centrifuged at 500 × *g* for 30 min at 4°C. Cells at the interface were collected, washed twice in L-15 medium containing P/S and 5% FCS, and adjusted to 2 × 10^6^ cells in the same media.

### Flow Cytometry and Cell Sorting

The anti-trout IgM [1.14 mAb mouse IgG1 coupled to fluorescein isothiocyanate or to allophycocyanin (APC), 1 μg/ml], the anti-trout major histocompatibility complex (MHC) II β-chain (mAb mouse IgG1 coupled to APC, 2 μg/ml), and the anti-trout CD8 (mAb rat IgG coupled to phycoerythrin, 7 μg/ml) used in this study have been previously characterized (23). Leukocytes were incubated with specific antibodies for 30 min on ice, washed three times with staining buffer (L-15 without phenol red containing 1% FCS), counterstained with 0.2 μg/ml 4′,6-diamidino-2-phenylindole (Sigma), to remove dead cells, and analyzed. In all cases, isotype controls for mouse mAbs were tested in parallel to discard unspecific binding of the Abs (fluorescein isothiocyanate-mouse IgG_1_ and APC-mouse IgG_1_, Biolegend). All samples were analyzed on a FACS Celesta™ flow cytometer (BD Biosciences) equipped with BD CellQuest™ Pro software following the gating strategy described in Figure S1A. In all cases, 20,000 events (live single cells) were acquired per sample. FACS sorting of IgM^+^ B cells, splenic T cells, or skin CD8^+^ dendritic cells (DCs) was performed on a FACSAria™ III flow cytometer (BD Biosciences) equipped with BD FACSDiva™ software as previously described (23, 24). The purity of the sorted samples was verified by flow cytometry as described in Figure S1B. All flow cytometry analyses were performed with FlowJo v10 (FlowJo, LLC. TreeStar).

### Real-Time PCR

To evaluate the levels of transcription of different immune genes in sorted leukocyte populations, DNase I-treated total RNA was reverse transcribed directly from FACS sorted populations using the Power SYBR Green Cells-to-Ct Kit (Invitrogen) following the manufacturer's instructions. Real-time PCR was performed using SYBR Green PCR core Reagents (Applied Biosystems) using specific primers (Table S1) and following the manufacturer's instructions as previously described (23).

### Production of Recombinant Rainbow Trout CD40L

The nucleotide sequence corresponding to the extracellular domain of one of the two rainbow trout CD40L sequences present in the rainbow trout genome (GenBank Accession number NP_001118138) together with an N-terminal 6x histidine tag was synthetized and subcloned into the E3 expression vector (Abyntek). The recombinant plasmid was transformed into BL21 cells, and a kanamycin-resistant single positive colony was then incubated at 37°C in Luria–Bertani media. When the OD_600_ reached 0.6, 0.1 mM of isopropyl β-d-thiogalactoside (Sigma Aldrich) was added to induce protein production. After 16 h, cells were harvested, lysed by sonication, and dissolved using urea. Thereafter, CD40L was obtained through the use of nickel columns (Sigma Aldrich). The CD40L-containing fractions were pooled and refolded. For the refolding, 0.5 M l-arginine was added to 4.5 ml of CD40L, and the protein was then dialyzed into 450 ml of 50 mM Tris–HCl, 150 mM NaCl, 10% glycerol, and 0.5 M l-arginine, pH 9.0. The dialysis was performed for 4 h using a 14 kDa cut-off dialysis membrane. At this point, the buffer was changed, and the protein was dialyzed for an additional 16 h. After dialysis, the sample was centrifuged at 13,000 rpm for 30 min and filtered through a 0.22-μm filter and packaged aseptically. Protein concentration was determined in a bicinchoninic acid protein assay (Thermo Fisher Scientific), and the recombinant rainbow trout CD40L (0.4 mg/ml) was aliquoted and stored at −80°C until use. The ToxinSensor^TM^ Chromogenic LAL Endotoxin Assay Kit (GenScript Inc.) was used to confirm the absence of LPS in the recombinant protein. An irrelevant protein with a similar molecular weight to that of recombinant CD40L (24.5 kDa), also bearing an N-terminal His tag, was produced in the same conditions and used as a functional control in preliminary experiments (C-His). CD40L was used in all experiments at 5 μg/ml after having established this as an optimal dose in initial experiments (data not shown). This dose of soluble CD40L is within the range of soluble CD40L doses used in previous mammalian studies (25, 26).

### B Cell Proliferation

The Click-IT^®^ EdU Alexa Fluor^®^ 488 Flow Cytometry Assay Kit (Life Technologies) was used to measure the proliferation of IgM^+^ B cells following manufacturer's instructions. Briefly, splenocytes were incubated with TNP-KLH, TNP-LPS, TNP-Ficoll, or CD40L, or left untreated (control) for 72 h at 20°C. Thereafter, 1 μM EdU was added to the cultures, and the cells were incubated for an additional 24 h. At this point, the cells were collected and stained with APC-anti-IgM mAb. Cells were then fixed, permeabilized, and incubated with specific reagents to detect the incorporation of EdU to the DNA of proliferating cells following the manufacturer's instructions. Samples were then analyzed by flow cytometry as described above.

### Calcium Flux

For calcium flux analysis, the calcium indicator Fluo-3 AM (Life Technologies) was used, following the manufacturer's instructions. Briefly, Fluo-3 was dissolved in dimethyl sulfoxide (Sigma-Aldrich) and further diluted in an equal volume of 20% (*w*/*v*) Pluronic^®^ F-127 (Life Technologies). Splenocytes were cultured in the presence or absence of TNP-KLH, TNP-LPS, TNP-Ficoll, or CD40L during 48 h. After the incubation period, cells were diluted in L-15 medium without FCS and incubated with Fluo-3 AM at a final concentration of 5 μM for 1 h. Cells were then collected and washed and a baseline reading for 30 s acquired in a FACSCalibur™ flow cytometer. Then, 0.5 μg/ml of APC-labeled anti-IgM were added to the tube and the emission of fluorescence (525 nm) determined for 180 s in gated IgM^+^ B cells from each sample following the strategy described in Figure S1C.

### ELISPOT

ELISPOT was used to quantify the number of total IgM-secreting B cells. For this, splenocytes were incubated with TNP-KLH, TNP-LPS, TNP-Ficoll, or CD40L, combinations of TNP antigens with CD40L, or left untreated (control) for 48 h at 20°C. Thereafter, cells were transferred to ELISPOT plates previously coated with an anti-IgM mAb and incubated for a further 24 h as previously described (23). Leukocytes from individual fish were added to the wells in triplicate at a concentration of 5 × 10^4^ cells per well. After 24 h of incubation at 20°C, cells were washed away five times with phosphate-buffered saline (PBS), and plates were blocked again with 2% bovine serum albumin in PBS for 1 h at room temperature (RT). After blocking, biotinylated anti-trout IgM mAb (clone 4C10) was added to the plates (1 μg/ml) and incubated for 1 h at RT. Following additional washing steps (five times in PBS), the plates were developed as previously described (23) and the number of spots in each well-determined using an AID iSpot Reader System (Autoimmun Diagnostika GMBH).

### Antigen Processing Capacity

To quantify the impact of CD40L on the capacity of IgM^+^ B cells to process antigens, the EnzChek protease Assay kit (Invitrogen) was used. For this, splenocytes were incubated with recombinant CD40L or left unstimulated during 72 h at 20°C. After this time, the cells were incubated with green fluorescent BODIPY DQ-casein at 5 μg/ml during 1 h. Afterwards, the cells were washed with FACS staining buffer three times and labeled with APC-anti-IgM mAb for 30 min at 4°C, washed again, and analyzed by flow cytometry as described above.

### RNA Extraction, Library Construction, and Sequencing

Total RNA was extracted from 1 × 10^5^ FACS isolated IgM^+^ B cells from control cultures or cultures stimulated with TNP-LPS or TNP-KLH (5 μg/ml) for 24 h using the RNAeasy micro kit (Qiagen). RNA concentration and RNA integrity (RIN) of each sample were measured on an Agilent 2100 Bioanalyzer (Figure S2). Total RNAs isolated from rainbow trout B cells showing RIN higher than 7 were used for library preparation and sequencing (Figure S2). The RNA-seq library preparation and sequencing was carried out by ZF-Genomics (Netherlands). Briefly RNA-seq libraries were prepared using the Truseq stranded total RNA kit (Illumina) including a unique index per sample. Libraries were pooled and sequenced together on an Illumina HiSeq 2500 sequencing system using single-read 50 bp (SR50) chemistry with a read depth of >10 million reads/sample.

### Short Reads Processing and Differential Expression Analysis

Raw sequence reads which passed Illumina quality controls were demultiplexed and adaptor trimmed using the Illumina pipeline. Reads from nine individual samples were mapped against the *O. mykiss* genome (27) available at http://www.genoscope.cns.fr. Mapping was performed using TopHat software on genome file *Oncorhynchus_mykiss*_chr_annot.gff adjusting parameters to a minimum intron length of 50, using a very sensitive option, the option coverage-based search for junctions disabled, and with the rest of option in default parameters. No mapped reads were ignored in successive analysis. Total read counts for each gene was merged using Samtools (28) and HTSeq for python (29). Read count normalization and differential expression analysis were performed using DESeq software (30). Three pair comparison were carried out, TNP-KLH stimulated IgM^+^ B cells vs. unstimulated IgM^+^ B cells, TNP-LPS stimulated IgM^+^ B cells vs. unstimulated IgM^+^ B cells, and TNP-LPS vs. TNP-KLH stimulated IgM^+^ B cells. A gene was considered as differentially expressed when the *p*-value adjusted for multiple testing with the Benjamini–Hochberg procedure (31) was <0.05. A normalized read count matrix was used to perform hierarchical clustering analysis using the Multiexperiment Viewer software (v4.9.0) (32). Genes were row normalized, and hierarchical clustering was constructed using Pearson correlation and the average linkage clustering method. Venn diagrams were constructed using the BioVenn software.

### Protein Assignment and Functional Annotation of Differentially Expressed Genes

Nucleotide sequences from genes showing expression were used as query for a BLASTx against two set of proteins. A comparison with RefSeq proteins from *O. mykiss* was carried out to assign the most updated protein description for the species. In parallel, a comparison with RefSeq proteins from a set of model species (*Homo sapiens, Mus musculus, Danio rerio, Macata mulata, Drosophila melanogaster*, and *Xenopus tropicalis*) was performed applying as threshold a minimum *E* value of 10^−5^. BLASTx results from this comparison were conducted with Blast2GO software (version V.2.7.2) (33) to identify and infer functional annotations including Gene Ontology (GO) terms from the three different GO ontologies categories (biological process, molecular function, and cellular component) as well as information relative to enzyme codes involve in metabolic pathways from Kyoto Encyclopedia of Genes and Genomes (KEGG). In addition, InterProScan software implemented in Blast2GO was used to compare sequences against protein families, domains, and functional sites from secondary databases, which were also used to infer functional annotation. Protein description was used to identify differentially expressed immune-relevant genes, which were grouped in PRRs; tumor necrosis factor superfamily (TNFSF), tumor necrosis factor and its soluble receptor (TNFSR), and related proteins, chemokines and chemokines receptors; cytokines, cytokine receptors, and related proteins; and interferon (IFN)-related proteins.

### Single Enrichment Analysis

Single enrichment (SE) analysis was performed in two different ways. On the one hand SE was carried out using GO term annotations previously assigned to differentially expressed genes with Blast2GO. GO term enrichment was analyzed using FatiGO (34) implemented in Blast2GO software. This program conducts a Fisher's exact test for 2 × 2 contingency tables to check for significant overrepresentation of GO annotations. GO term enrichment was evaluated by comparison of GO terms identified within genes overrepresented in stimulated IgM^+^ B cells vs. GO terms overrepresented in unstimulated IgM^+^ B cells for TNP-LPS and TNP-KLH stimulations. A GO term was considered significantly enriched when the *p* < 0.05. On the other hand, to be able to use molecular information from human databases, the best blast hit identified within *H. sapiens* proteins according BLASTx results, when occurred, was assigned to an *O. mykiss* gene. The protein accession number or the equivalent Entrez Gene ID was finally assigned using gene_info file from National Center for Biotechnology Information Gene FTP directory. Putative protein–protein interaction and KEGG pathways enrichment analysis was performed inferring human homology throughout String database (35). A pathway was considered significantly enriched when the *p*-value adjusted for multiple testing was <0.05. Human Entrez Gene IDs previously identified were also used for mapping genes against the different pathways from KEGG. Expression values from different genes identified along pathways were highlighted using Pathview web software (36).

### Statistical Analysis

Statistical analyses were performed using an ANOVA followed by a two-tailed Student's ***t***-test with Welch's correction when the *F* test indicated that the variances of both groups differed significantly. The differences between the mean values were considered significant on different degrees, where ^*^ means *p* ≤ 0.05, ^**^ means *p* ≤ 0.01, and ^***^ means *p* ≤ 0.005.

## Results

### Effect of CD40L on Teleost B Cells

**Figure 1:**
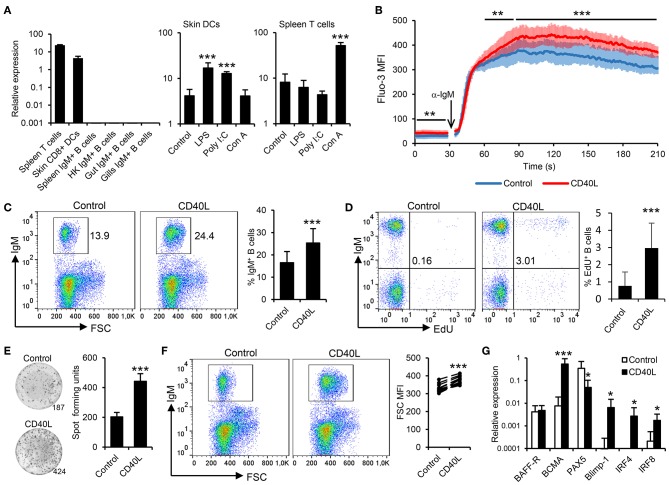
Trout IgM^+^ B cells are highly responsive to CD40L. **(A)** CD40L transcription levels were analyzed through real-time PCR in rainbow trout FACS-isolated leukocyte subsets. In some cases, cells were previously incubated for 18 h with lipopolysaccharide (LPS), poly I:C, concanavalin A (ConA), or with media alone (control). The relative expression to the endogenous control EF-1α was calculated and is shown as mean + SD (*n* = 6). Spleen leukocytes (*n* = 12) were incubated with CD40L (5 μg/ml) or control media alone at 20°C and diverse assays performed at different times poststimulation. **(B)** After 48 h, calcium flux was assayed upon BCR stimulation. Data are represented as mean fluorescence intensity (MFI) (solid line) ± SD (shaded areas) of intracellular Ca^2+^ levels in IgM^+^ B cells. **(C)** After 72 h, the percentage of live IgM^+^ B cells among the lymphocyte gate was determined. Representative dot plot and mean IgM^+^ B cell survival + SD are shown. **(D)** After 72 h, cells were labeled with EdU and incubated for a further 24 h. The percentage of proliferating (EdU^+^) IgM^+^ B cells were then determined. Representative dot plot and mean percentage of proliferating B cells within the IgM^+^ compartment + SD are shown. **(E)** After 72 h, an ELISPOT was conducted. Representative wells and quantification of spot forming cells are shown as mean + SD. **(F)** The size of the IgM^+^ B cell population was also measured after 72 h and calculated as MFI of FSC. Representative dot plot and MFI of FSC for each individual fish are shown. **(G)** After 24 h, IgM^+^ B cells were FACS isolated and RNA extracted to determine the transcription of specific genes relative to EF-1α. Results are shown as mean relative expression + SD. In all experiments, statistical differences were evaluated by a one-way ANOVA followed by a two-tailed Student's *t*-test, where ^*^p ≤ 0.05, ^**^p ≤ 0.01, and ^***^p ≤ 0.005.

Rainbow trout contain two different sequences in their genome that seem to correspond to CD40L homologs, sharing an 81.44% amino acid identity (Figure S3). Based on these results, in the current study, we focused on determining the levels of expression and the effects of one of these CD40L which was previously reported to be highly expressed in rainbow trout CD4^+^ T helper cells (37). Before determining the effect of CD40L on rainbow trout IgM^+^ B cells, we verified which leukocyte subsets were expressing this CD40L in this species. As expected, splenic T cells showed the highest transcription of CD40L, closely followed by skin CD8^+^ DCs, a subset of rainbow trout DCs recently identified (24). In contrast, CD40L transcription was not detected on resting IgM^+^ B cells from diverse sources (Figure 1A). To further investigate whether DCs and T cells had the capacity to regulate CD40L transcription upon activation, we incubated the corresponding leukocyte cultures with different activation signals and then FACS isolated DCs or T cells to determine CD40L transcription levels. Our results show that skin DCs significantly upregulated the expression of CD40L in response to LPS or poly I:C, while T cells upregulated the expression of CD40L in response to the T cell mitogen concanavalin A (ConA) (Figure 1A).

To date, the effects of CD40L on teleost B cells have only been indirectly demonstrated (38). Thus, we generated rainbow trout recombinant CD40L and analyzed its function on trout splenic IgM^+^ B cells, following the gating strategy shown in Figure S1, acquiring 20,000 singlet live cells in all samples. First, we determined whether CD40L stimulation could influence the calcium response to BCR cross-linking. After 48 h of incubation with CD40L, the calcium flux triggered after BCR engagement was significantly increased on IgM^+^ B cells when compared to untreated cells (Figure 1B). In addition, incubation of splenocytes cultures with CD40L provoked a significant accumulation of IgM^+^ B cells after 72 h (Figure 1C), since IgM^+^ B cells accounted for ~16% in untreated splenocyte cultures, while they constituted more than 25% in CD40L-stimulated cultures. To clarify whether this accumulation was a consequence of CD40L-mediated increased B cell survival or proliferation, proliferation assays were conducted that verified that CD40L alone was capable of inducing the proliferation of a significant subset of IgM^+^ B cells (Figure 1D).

We next determined whether CD40L by itself was also able to elicit the differentiation of B cells into IgM-secreting cells, namely, plasmablasts or plasma cells. To this end, we performed an ELISPOT assay, which revealed that CD40L significantly augmented the number of IgM-secreting cells in splenocyte cultures after 3 days (Figure 1E). As expected during a B cell differentiation process, the increase in the number of IgM-secreting cells was accompanied by a significant increase in cell size, as determined by flow cytometry (Figure 1F). This general increase in cell size correlated with a significant increase in the percentage of IgM^+^ B cells cataloged as large cells (Figure S4). All these results, pointed to a CD40L-mediated differentiation program to plasmablasts/plasma cells. To further confirm this, we compared the levels of transcription of several marker genes in both CD40L-stimulated and untreated IgM^+^ B cells. Our results show that in response to CD40L, IgM^+^ B cells significantly downregulated the expression of the transcription factor Pax5 and upregulated the messenger RNA (mRNA) levels of the transcription factors Blimp1 and IRF4 as well as those of the B cell maturation antigen (Figure 1G). In mammals, this transcriptional profile is indicative of a terminal differentiation of B cells to plasma cells (39–41). Surprisingly, whereas IRF4 is involved in plasma cell differentiation, in mammals, it antagonizes with IRF8 during the B cell differentiation process, as IRF8 promotes the differentiation of B cells to GC B cells (41). In rainbow trout, CD40L upregulated both IRF4 and IRF8 transcription (Figure 1G), suggesting that there could be differences in the role that these factors play during the B cell differentiation process, although these regulations should also be verified at protein level.

**Figure 2:**
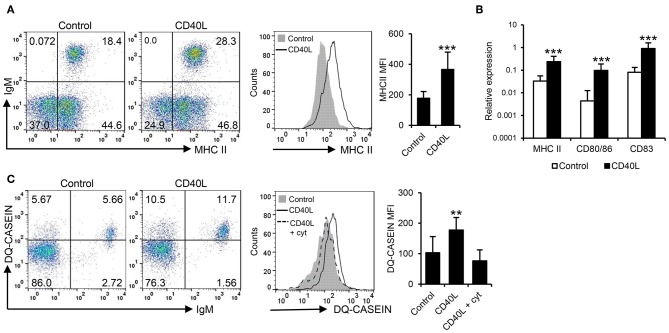
CD40L regulates the antigen presenting capacities of trout IgM^+^ B cells. Spleen leukocytes (*n* = 12) were incubated with CD40L (5 μg/ml) or control media alone at 20°C and different assays performed at different times poststimulation. **(A)** After 72 h, cells were labeled with anti-trout IgM and anti-trout MHC II mAbs. Representative dot plots and histogram showing MHC II expression levels on IgM^+^ B cells from one fish is shown together with a quantification of major histocompatibility complex II (MHC II) mean fluorescence intensity (MFI) values, shown as mean + SD. **(B)** After 24 h, IgM^+^ B cells were FACS isolated and the transcription levels of MHC II, CD80/86, and CD83 relative to EF-1α calculated for each sample and shown as mean + SD. **(C)** After 72 h, the capacity of IgM^+^ B cells to process DQ-casein was also analyzed by flow cytometry. Representative dot plots and histogram and quantification shown as mean + SD are included. In all experiments, statistical differences were evaluated by a one-way ANOVA followed by a two-tailed Student's *t*-test, where ^**^p ≤ 0.01 and ^***^p ≤ 0.005.

In mammals, CD40L also has the capacity to increase the antigen presenting capacities of B cells (42). Hence, we investigated the effects of CD40L on the antigen processing and presentation capacities of fish IgM^+^ B cells. Our results clearly show that CD40L significantly increased the levels of surface MHC II on IgM^+^ B cells (Figure 2A). In correlation with these results, CD40L was also shown to increase the transcription levels of not only MHC II but also of the costimulatory molecules CD83 and CD80/86 (Figure 2B). Furthermore, CD40L significantly augmented the capacity of fish IgM^+^ B cells to process antigens, as the percentage of cells able to process DQ-casein, a self-quenched form of fluorescently labeled casein, significantly increased after treatment with CD40L, as did the mean fluorescence intensity (MFI) derived from the degradation of the antigen (Figure 2C). This effect was blocked when cells were coincubated with CD40L in the presence of cytochalasin D, known to inhibit the antigen processing machinery in B cells (43).

### Effect of TD and TI Antigens on Teleost B Cell Activation

**Figure 3:**
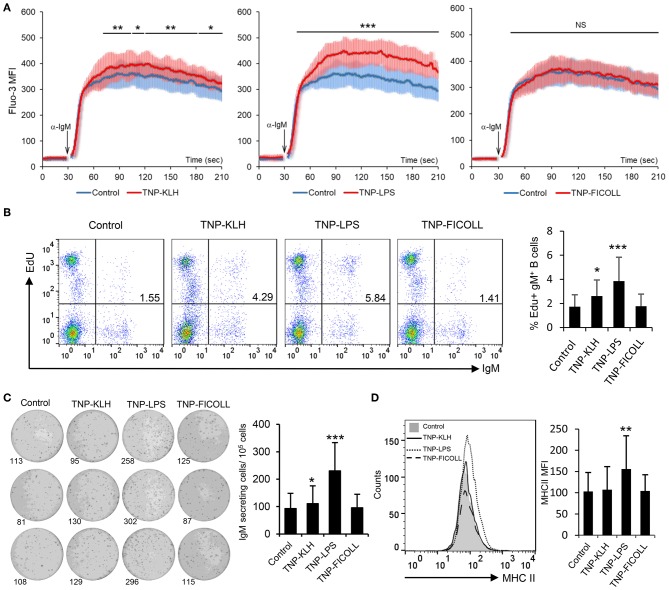
Effect of thymus-dependent (TD) and thymus-independent (TI) antigens on teleost B cell activation. Spleen leukocytes (*n* = 6) were incubated with 2,4,6-trinitrophenyl hapten conjugated to keyhole limpet hemocyanin (TNP-KLH), TNP conjugated to lipopolysaccharide (TNP-LPS), and TNP-Ficoll (5 μg/ml) or left unstimulated (control) at 20°C and different assays performed at different times poststimulation. **(A)** After 48 h, calcium flux was assayed upon B cell receptor (BCR) stimulation. Data are represented as mean fluorescence intensity (MFI) (solid line) ± SD (shaded areas) of intracellular Ca^2+^ levels in IgM^+^ B cells. **(B)** After 72 h, cells were labeled with EdU and incubated for a further 24 h. The percentages of proliferating (EdU^+^) IgM^+^ B cells among the total lymphocyte population were then determined. Representative dot plot and mean percentage of proliferating B cells within the IgM^+^ compartment + SD are shown. **(C)** After 72 h, an ELISPOT was conducted. Representative wells and quantification of spot forming cells are shown as mean + SD. **(D)** After 72 h, cells were labeled with anti-trout IgM and anti-trout major histocompatibility complex II (MHC II) mAbs. Representative histogram showing MHC II expression levels on IgM^+^ B cells from one fish is shown together with a quantification of MHC II MFI values, shown as mean + SD. Statistical differences were evaluated by a one-way ANOVA followed by a two-tailed Student's *t*-test, and asterisks denote significant differences in stimulated cultures compared to control cultures, where ^*^p ≤ 0.05, ^**^p ≤ 0.01, and ^***^p ≤ 0.005. NS, nonsignificant.

Having established that rainbow trout splenic IgM^+^ B cells strongly respond to CD40L stimulation, we decided to evaluate how these cells responded to antigens defined as TD or TI on the basis of their capacity to stimulate B cells in the absence of T cell help. To carry this out, we used TNP-KLH as a TD model antigen and TNP-LPS and TNP-Ficoll as TI-1 and TI-2 model antigens, respectively. Of course, it has to be taken into consideration that the classification of antigens as TI-1 and TI-2 is not only based on their structure but also on the effects they exert on mammalian models, still not knowing whether their mechanisms through which they signal are the same in teleost fish. After incubating splenocytes with either of these antigens for 48 h, we observed a strong increase in the calcium flux triggered by BCR engagement in cells that had been previously exposed to TNP-LPS when compared to untreated cells (Figure 3A). This increased responsiveness of the BCR was also observed to a lower extent on TNP-KLH-treated cells but was never shown in cells stimulated with TNP-Ficoll (Figure 3A). We next analyzed the capacity of the different antigens to promote the proliferation of splenic IgM^+^ B cells. Paralleling the results observed in the calcium flux assays, a significantly increased proliferation of IgM^+^ B cells in response to TNP-LPS was clearly visible (Figure 3B). Although significant, the lymphoproliferative capacities of TNP-KLH were much lower, while no proliferation was observed in response to TNP-Ficoll (Figure 3B). To assess whether these antigens could induce the differentiation of rainbow trout IgM^+^ B cells, we performed an ELISPOT assay after 3 days of incubation. In this case, only TNP-LPS was capable of significantly augmenting the number of IgM-secreting cells in the cultures, whereas no effect was seen in response to TD or TI-2 antigens (Figure 3C). Similarly, only TNP-LPS was capable of increasing the levels of surface MHC II expression on IgM^+^ B cells (Figure 3D and Figure S5). These data suggest that TI-1 antigens exhibit superior capacities to activate and promote the differentiation of trout IgM^+^ B cells.

### Transcriptomic Responses to TI-1 and TD Antigens of Isolated Splenic IgM^+^ B Cells

**Figure 4:**
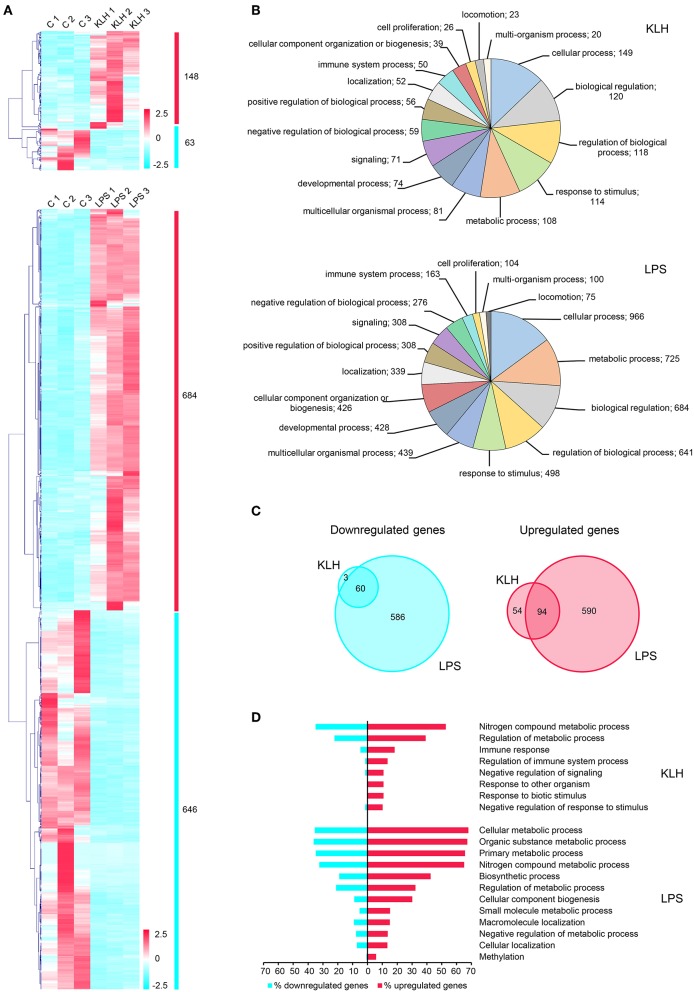
Global analysis of differentially expressed genes in TNP-LPS and TNP-KLH stimulated B cells. **(A)** Clustering analysis of differentially expressed genes in TNP-LPS and TNP-KLH stimulated IgM^+^ B cells. Genes were row normalized and hierarchical clustering constructed using Pearson correlation and the average linkage clustering method. **(B)** Pie chart showing the number of differentially expressed genes in TPN-KLH or TNP-LPS annotated with different Gene Ontology (GO) terms at level 2 of biological process category. **(C)** Venn diagram showing the number of upregulated and downregulated genes for each treatment. Intersections show the number of differentially expressed genes shared between both stimulations. **(D)** Single enrichment analysis between upregulated and downregulated genes for each treatment. Bars show the percentage of genes identified for each GO term at level 3 from biological process category, which showed a significant enrichment (*p* < 0.05) in upregulated genes.

To further investigate the differential effects exerted by TD and TI-1 antigens on trout IgM^+^ B cells, RNA sequencing (RNA-seq) was performed on FACS isolated splenic IgM^+^ B cells that had been stimulated with TNP-KLH or TNP-LPS for 24 h, or on control unstimulated splenic IgM^+^ B cells. Total RNAs isolated from rainbow trout IgM^+^ B cells showing RNA integrity numbers (RIN) higher than 7 according to the bioanalyzer results were used for library preparation and sequencing (Figure S2). A total of 150 million reads were obtained, with an average of 16.6 million reads per sample. After mapping a total of 87 million reads against *O. mykiss* genome, genes reaching five reads in at least one sample were maintained, identifying 31,691 genes expressed in *O. mykiss* IgM^+^ B cells. To investigate which cellular processes were triggered in IgM^+^ B cells in response to stimulation, a differential expression analysis was performed between stimulated and unstimulated IgM^+^ B cells, as well as between cells stimulated with the different antigens. DESeq results highlighted a total of 1,441 genes showing significant differences in their transcript levels in at least one of the comparisons carried out (Figure 4A and Table S2). Interestingly, the fact that several genes show the same description seems to correspond to homolog genes originated after the whole-genome duplication event that took place in salmonids within the last 100 million years, as previously reported (44). Although some of the homolog genes generated through this late duplication event have been demonstrated to have diverged slightly in their function (44), it should be noted that they usually showed a high correlation in gene expression (Table S2), as previously reported during analysis of the rainbow trout genome (27).

As expected from the results obtained in our functional studies, TNP-LPS provoked transcriptional changes in a larger number of genes when compared to TNP-KLH (Figure 4A). Thus, a total of 1,330 genes were significantly modulated in TNP-LPS-stimulated IgM^+^ B cells when compared to unstimulated IgM^+^ B cells, identifying 684 upregulated and 646 downregulated genes (Figure 4A). In contrast, TNP-KLH provoked transcriptional changes in a lower number of expressed genes, with only 148 upregulated and 63 downregulated genes when compared to unstimulated IgM^+^ B cells (Figure 4A). A functional annotation in GO terms was assigned to differentially expressed transcripts using Blast2GO software, allocating one biological process category (level 2) to each transcript. This analysis revealed that 50 genes among those differentially expressed in response to TNP-KLH were annotated as related to “immune system process,” while 163 genes differentially expressed upon TNP-LPS stimulation fell into this category (Figure 4B). Other differentially expressed genes were ascribed to categories such as metabolic process, cellular process, biological regulation, or response to stimulus. In all these cases, the number of genes differentially expressed in response to TNP-LPS in each category was always higher than that observed in response to TNP-KLH (Figure 4B). Despite the strong divergence between the responses to the two stimuli, 60 genes were commonly downregulated and 94 commonly upregulated in response to both TNP-LPS and TNP-KLH (Figure 4C and Table S2). Single enrichment analysis further highlighted several functionalities that were significantly increased in both treatments (Figure 4D). Whereas, the stimulation with TNP-LPS resulted in a significant increase in genes involved in general cell metabolism identifying GO terms as “cellular metabolic process,” “organic substance metabolic process,” “primary metabolic process,” “nitrogen compound metabolic process,” or “biosynthetic process,” the stimulation with TNP-KLH showed an enrichment in GO terms related to “immune response,” “regulation of immune system process,” “negative regulation of signaling,” or “response to biotic stimulus” (Figure 4D). These results suggest that although TNP-KLH is able to regulate a group of genes related to the recognition of the stimulus and subsequent immune response, it is not capable to trigger on its own the complete activation/differentiation transcriptomic profile that is achieved in response to TNP-LPS that implies an extensive alteration of the cellular metabolism. However, it is interesting to highlight that 57 genes significant modulated in response to TNP-KLH were not significantly modified by TNP-LPS (54 upregulated and 3 downregulated) (Table S3), demonstrating that the lower effects of TNP-KLH on trout IgM^+^ B cells are not a result of an insufficient stimulation, but rather of a differential response.

### Analysis of Differentially Expressed Immune Genes in Isolated IgM^+^ B Cells Stimulated With TI-1 or TD Antigens

**Figure 5:**
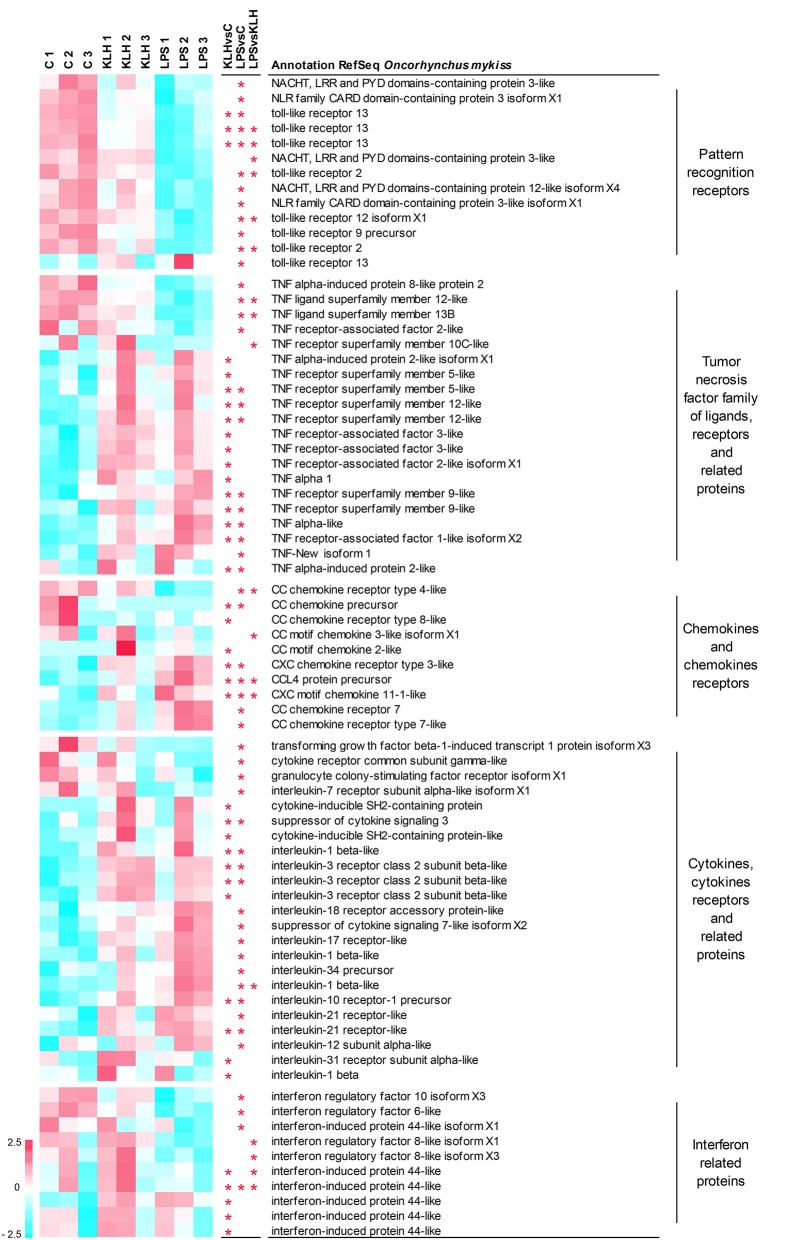
Clustering analysis of differentially expressed genes in TNP-LPS and/or TNP-KLH stimulations related to different immune-relevant groups. Genes were row normalized and hierarchical clustering constructed using Pearson correlation and the average linkage clustering method. Genes were grouped in pattern recognition receptors; tumor necrosis factor family of ligands, receptors, and related proteins; chemokines and chemokines receptors; cytokines, cytokine receptors, and related proteins; and interferon related proteins. Central columns highlight genes showing significant differential expression (FDR *p* < 0.05) when TNP-KLH was compared to Control (KLHvsC), TNP-LPS to Control (LPSvsC), or TNP-LPS to TNP-KLH (LPSvsKLH).

As stated before, many of the genes differentially regulated in IgM^+^ B cells in response to TNP-LPS or TNP-KLH corresponded to genes that code for elements of the immune system (Table S2). Among them, we focused on those that are particularly relevant to B cell activation processes, namely pattern-recognition receptors; members of the TNFα family of ligands and receptors and associated molecules; chemokines and chemokine receptors; cytokines, cytokine receptors, and related proteins; and interferon (IFN) related proteins (Figure 5).

A total of 13 genes among those differentially expressed in response to either TNP-LPS or TNP-KLH in comparison to untreated cells were identified as pattern-recognition receptors, including eight sequences identified as TLRs and five sequences recognized as NOD-like receptors (Figure 5). Nearly all of these genes showed a significant decrease in their transcript levels upon TNP-LPS stimulation that was not observed in response to TNP-KLH (Figure 5). Given the suggested lack of TLR4 in salmonids, the TLR(s) responsible for the sensing of LPS has not yet been identified in these species (45); however, these results seem to confirm that PRR signaling routes are significantly altered by TI-1 antigens and not by TD antigens.

It has been extensively described that members of the TNF superfamily of ligands (TNFSF) and receptors (TNFRSF) play key roles on the homeostasis and activation of B cells both in mammals (46) and fish (47). Among TNFSF, the transcription levels of tnfsf12 (TWEAK) and tnfsf13b (BAFF) were significantly downregulated upon TNP-LPS stimulation but not in response to TNP-KLH (Figure 5). By contrast, the transcription of one TNFα homolog (TNFα1) was induced in response to TNP-KLH, whereas another homolog of TNFα (TNFα3) (48) was significantly upregulated in IgM^+^ B cells in response to both stimuli. Finally, TNF-New, proposed as a homolog of mammalian lymphotoxin-beta (LTβ) (49), was only significantly upregulated by TNP-LPS. Regarding the receptors, the mRNA levels of one gene encoding tnfrsf5 (CD40), two genes encoding two copies of tnfrsf9 (4-1BB), and two genes encoding two copies of tnfrsf12 (TWEAK receptor, TWEAKR) were significantly augmented after either TNP-LPS or TNP-KLH stimulation (Figure 5). Interestingly, an additional CD40 gene was exclusively upregulated in response to TNP-KLH (Figure 5). Significant differences were also observed in tnfrsf10C (TRAIL receptor3, TRAILR3) expression levels in response to the different stimuli, showing an opposite trend when compared to unstimulated cells. TNF receptor-associated factors (TRAFs) are essential for the transduction of many receptors implicated in cellular immune responses (50). There are six known TRAF family members (TRAF1 to 6) in mammals. Although originally identified for their implication in the signal transduction of TNFRSF members, it was later demonstrated that they are also mediators of the intracellular signaling cascade of many receptors, including TLRs, NOD-like receptors or IFN receptors (51). Our results revealed a differential upregulation of TRAF1, TRAF2, and TRAF3 in response to IgM^+^ B cell stimulation. While TRAF1 was significantly upregulated in both TNP-LPS- or TNP-KLH-stimulated IgM^+^ B cells, TRAF2 and TRAF3, implicated in the transduction of CD40L signaling, were exclusively upregulated in response to TNP-KLH (Figure 5). Interestingly, a second gene ascribed as TRAF2 presented an opposite trend, as its transcription was significantly decreased after TNP-LPS stimulation (Figure 5).

Among cytokines, chemokines and their receptors were also importantly regulated in trout IgM^+^ B cells response to stimulation. In fish, it is quite difficult to establish true orthologs between fish chemokines and mammalian chemokines due to the extensive duplication events that the fish genome has suffered and because chemokines are proteins that have rapidly evolved in response to the different antigenic experiences (52). This is especially true for CC chemokines. Some of these sequences that correspond to CC chemokines were transcriptionally downregulated in response to stimulation, whilst others such as CCL4 were upregulated (Figure 5). CXCL11 was the only CXC chemokine significantly modulated upon stimulation in IgM^+^ B cells (Figure 5). In this case, both TNP-LPS and TNP-KLH induced its transcription, although the levels reached in response to TNP-LPS were significantly higher than those obtained upon TNP-KLH stimulation (Figure 5). Among chemokine receptors, CCR4 mRNA levels were significantly downregulated by TNP-LPS, whereas CCR8 transcription was only downmodulated in TNP-KLH-stimulated IgM^+^ B cells (Figure 5). In contrast, two genes that encode homologs of CCR7 exhibited a significant upregulation in TNP-LPS stimulated IgM^+^ B cells, while a gene encoding CXCR3 significantly increased its transcript levels in response to both stimulations (Figure 5).

Other cytokines and cytokine receptors were further modulated in trout IgM^+^ B cells upon stimulation. Most of these genes were exclusively modulated in response to TNP-LPS, including genes that were downregulated such as transforming growth factor β, a colony-stimulating factor receptor or interleukin 17 receptor (IL17R), and genes that were upregulated such as IL1β, IL12, IL34, and another gene ascribed as IL7R (Figure 5). On the other hand, only a few genes were transcriptionally regulated in response to TNP-KLH exclusively, among which an IL3R and IL31R could be identified along with another gene copy of IL1β (Figure 5). Another subset of cytokine and cytokine receptor genes was significantly upregulated at a transcriptional level in response to both stimuli. These included a further IL1β gene, IL10R, and IL21R and two genes ascribed as IL3R (Figure 5). Some genes encoding different members of the suppressor of cytokine signaling family (SOCS) were cataloged in this group. Among them, two genes encoding CISH were significantly upregulated during the response to TNP-KLH, whereas SOCS3 was induced in both stimulations and SOCS7 specifically in response to TNP-LPS stimulation.

Finally, an important number of genes modulated in IgM^+^ B cells corresponded to IFN-regulatory factors (IRFs) or IFN-induced proteins. In most cases, these genes were transcriptionally downregulated in response to stimulation, either TNP-LPS (IRF6, IRF10) or TNP-KLH (IRF44). Interestingly, IRF8 transcription levels were significantly different between cells stimulated with TNP-LPS and TNP-KLH, showing opposite trends when compared to unstimulated controls (Figure 5).

### Analysis of Significantly Enriched Immune-Relevant Pathways in IgM^+^ B Cells in Response to Antigen Stimulation

**Figure 6:**
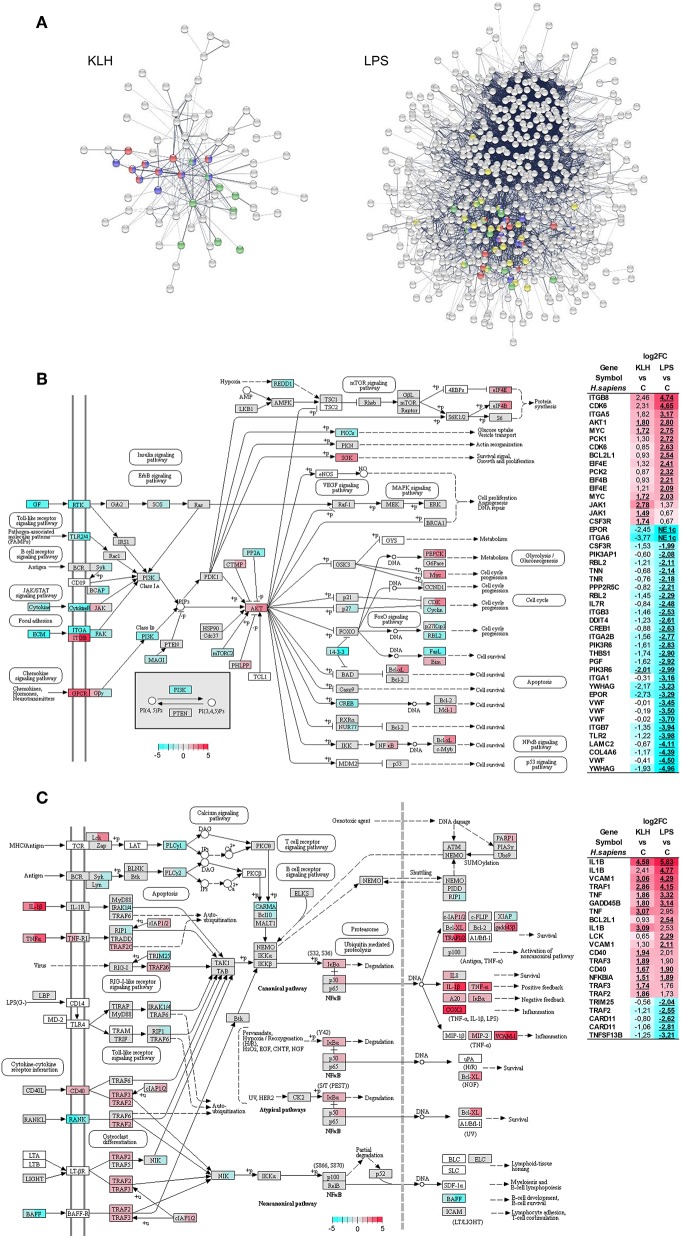
Protein–protein interaction and pathways analysis. **(A)** Protein–protein interaction network highlighting proteins from pathways related to immune system which were identified with significant enrichment within differentially expressed genes from TNP-KLH or TNP-LPS stimulated IgM^+^ B cells. Dark blue, TNF signaling pathway; red, NF-κB signaling pathway; green, Jak-STAT signaling pathway; yellow, PI3K-Akt signaling pathway. **(B)** Overview of the PI3K-Akt signaling pathway. **(C)** Overview of the NF-κB signaling pathway. Enzymes showing mapped reads during the experiment are highlighted according log_2_FC. Enzymes without identified homologs in *O. mykiss* are shown as white. For each node, the first section of square indicates the log_2_FC value observed in response to TNP-KLH stimulation, whereas the second section of square shows the log_2_FC value observed in response to TNP-LPS. When several genes were identified for one node, the gene with maximum absolute value was selected. To the right, differentially expressed genes (FDR *p* < 0.05) are listed. The color gradient represents highly upregulated (red) to highly downregulated (cyan blue) genes. The exact log_2_FC values are given for each gene and “NE 1c” is indicated when no mapped reads were identified for one condition.

Protein–protein interaction analysis using *O. mykiss* differentially expressed genes based in the homology with human proteins highlighted a significant enrichment in different KEGG pathways (Figure 6A). Thus, signaling pathways such those of TNFSF, nuclear factor κB (NF-κB) and Jak-STAT exhibited significant enrichments in response to both stimuli, whereas others such as the phosphoinositide 3-kinase (PI3K)-Akt signaling pathway were only significantly enriched in TNP-LPS-stimulated IgM^+^ B cells (Figure 6A). This protein–protein interaction analysis again highlighted the intense response of IgM^+^ B cells to TNP-LPS that resulted in a regulation of a high number of interacting proteins involved in several processes commonly related to cellular metabolism (Figure 6A). In contrast, the moderate response of IgM^+^ B cells to TNP-KLH was focalized in immune-system-related pathways (Figure 6A).

The PI3K-Akt signaling pathway plays a crucial role in B cell activation, differentiation, and survival integrating receptor-mediated signaling with cell metabolism (53, 54). Interestingly, 43 genes that were differentially transcribed in response to TNP-LPS were mapped along this pathway, 30 being downregulated and 13 upregulated (Figure 6B). The most represented group of differentially regulated genes within this pathway correspond to genes encoding cognate extracellular matrix ligands (TNN, TNR, THBS1, VWF, LAMC2, and COL4A6), all of them strongly downregulated in response to TNP-LPS (Figure 6B). Within this pathway, additional genes related to protein synthesis (EIF4B and EIF4E), metabolism (PCK1 and PCK2), cell cycle progression (CDK6), or survival (BCL2L1) were significantly upregulated exclusively in response to TNP-LPS (Figure 6B). Interestingly, the serine/threonine-protein kinase Akt and the transcription factor Myc were significantly upregulated in both TNP-LPS and TNP-KLH stimulated B cells (Figure 6B).

Many genes regulated in response to either TNP-LPS or TNP-KLH belonged to the NF-κB signaling pathway (Figure 6C). Thus, in this case, both stimuli seemed to effectively modulate this pathway, as TNP-KLH upregulated the transcription of 13 of these genes, while TNP-LPS significantly increased the mRNA levels of 11 of these genes, while downmodulated 5 of them (Figure 6C). Similarly, in mammals, BCR signaling, CD40, and TLRs have the capacity to individually engage NF-κB during B cell activation (55). In our experiments, although both TNP-LPS and TNP-KLH significantly upregulated the transcription of one homolog of CD40, another CD40 homolog and the downstream factors TRAF2 and TRAF3 were only significantly upregulated in response to TNP-KLH (Figure 6C).

### CD40L Synergizes With TI-1 Antigens to Activate IgM^+^ B Cells *in vitro* and *in vivo*

**Figure 7:**
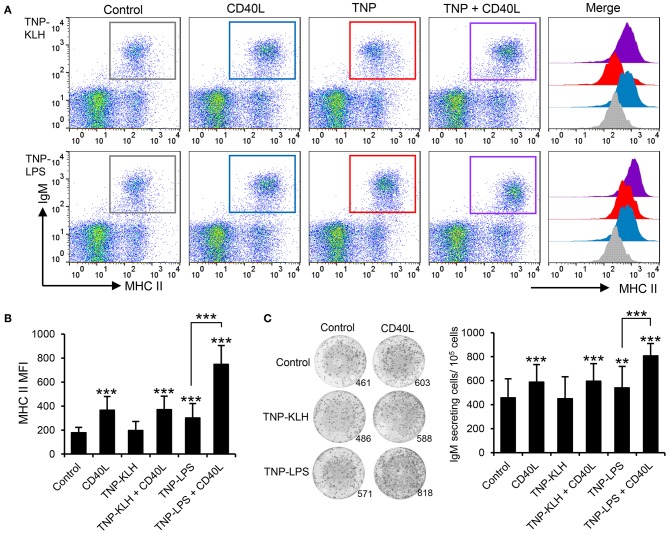
CD40L synergizes with TI-1 antigens to activate teleost IgM^+^ B cells. **(A)** Spleen leukocytes were incubated with CD40L (5 μg/ml), TNP-KLH (5 μg/ml), TNP-LPS (5 μg/ml), combinations of any of the TNP-bound antigens with CD40L, or left unstimulated (control) for 72 h at 20°C. After this time, cells were labeled with anti-IgM and anti-MHC II mAbs and analyzed by flow cytometry. Representative dot plots showing MHC II expression levels on IgM^+^ B cells from one representative fish are shown, in which IgM^+^ B cells were gated in control cells (gray), CD40L-treated cells (blue), TNP-antigen (red), and combination of CD40L plus TNP-antigen (purple). Histograms showing MHC II mean fluorescence intensity (MFI) on IgM^+^ gated B cells are also shown. **(B)** MFI values for surface MHC II were calculated on IgM^+^ B cells under the different treatments, and average values plotted as mean + SD (*n* = 12). **(C)** Spleen leukocytes were incubated as described above and after 72 h, an ELISPOT was conducted. Representative wells and quantification of spot forming cells are shown as mean + SD (*n* = 12). In all cases, statistical differences were evaluated by a one-way ANOVA followed by a two-tailed Student's *t*-test, where ^**^p ≤ 0.01 and ^***^p ≤ 0.005.

Taking into consideration the apparent contradiction of IgM^+^ B cells being able to respond to CD40L stimulation, but only being effectively activated by TI-1 antigens, we decided to perform a series of experiments to determine whether synergistic effects could be established between CD40L and the different antigens. Splenocytes were incubated with the different antigens in the presence or absence of CD40L and the levels of surface MHC II on IgM^+^ B cells studied through flow cytometry. A significant synergistic effect between CD40L and TNP-LPS was evident (Figures 7A,B). Similarly, the incubation of splenocytes with a combination of TNP-LPS and CD40L promoted the presence of a significantly higher number of plasma cells than that observed in response to TNP-LPS or CD40L alone (Figure 7C). Interestingly, these synergistic effects were never observed when CD40L was combined with TNP-KLH (Figures 7A–C).

## Discussion

In teleost fish, the fact that no cognate GCs are ever organized and there is no CSR seem to account for the low affinity maturation of antibodies during the primary and secondary immune responses observed in these species (56, 57). Furthermore, a substantial amount of evidence has been gathered by different authors in recent years pointing to a phenotypic and functional resemblance between fish IgM^+^ B cells and mammalian B1 cells (58–61). In this context, under the mammalian paradigm that long-lived memory responses are only achieved through TD class-switched responses mounted within GCs, it would seem plausible to predict that teleost fish are not able to mount long-lived B cell memory responses. However, studies performed many years ago by the group of Stephen Kaattari already revealed that teleost fish have the capacity to mount B cell memory responses to a certain extent (56, 62), demonstrating that a certain kind of memory-like B cells appeared in evolution before the acquisition of GCs. This teleost memory responses were already shown to mimic mammalian extrafollicular and IgM TI responses (56, 62), which have been recently described to be able to give rise to long-term immunological memory. For example, mice deficient in both T cells and follicular B cells, which exclusively bear B1 and MZ B cells, have been shown to be capable of effectively clearing a *Borrelia* infection through IgM (7, 8). Similarly, protection against *Ehrlichia muris* is also mediated by long-lived IgM secreting cells in the bone marrow (11). In addition, IgM^+^ long-lived PCs have also been identified in response to TD antigens in animals in which the formation of GCs was prevented (12). Thus, it now seems evident that B cell memory is composed of different layers (63), with a first layer of IgM memory B cells organized outside lymphoid follicles and a second layer of switched memory B cells shaped within GCs. Although these IgM responses should protect the organism until the second layer is fully organized, mutated IgM memory B cells were shown to persist even longer than IgG memory B cells, demonstrating their essential role in long-term protection (63). As previously suggested (13, 64), it is tempting to suggest that these layers are reminiscent of how evolution of the immune system has developed, with fish memory responses resembling this first layer of mammalian responses in which cells have the ability to persist but, in the absence of GCs, have not yet acquired the qualities to further enhance the efficiency of secondary responses.

In the current study, we have systematically explored the response of teleost B cells to different types of antigens and stimulatory signals confirming that in teleost, as recently described in different mammalian models, TD and TI stimulatory signals can add up to achieve different degrees of activation. First, we have verified through a wide range of techniques that fish IgM^+^ B cells respond poorly to TD antigens, while they are able to fully differentiate in response to TI-1 antigens. Despite this deficient response to TD antigens, our work shows that fish IgM^+^ B cells effectively respond to CD40L. This constitutes the first report demonstrating a B-cell-stimulating capacity for a teleost CD40L molecule. Interestingly, this capacity is present in fish despite the many structural differences between mammalian and teleost CD40L sequences reported (65). For example, five amino acids found essential in mammals for the interaction of CD40L with CD40 are not found in any teleost CD40L molecules (65, 66). Similarly, a glycosylation site conserved in mammalian, amphibian, and avian CD40L is not present in teleost CD40L sequences (65). Thus, our results demonstrate that regardless of the structural changes that this protein suffered through evolution, its main function was already present in teleost fish. Remarkably, as reported here and elsewhere (67, 68), CD40L can be provided by other cell types different than T cells, such as for example, DCs. Thus, accumulating evidence on the fact that DCs and other innate cell subsets such as invariant natural killer T cells are able to provide the second signal needed to activate B cells have compelled some researchers to differentiate between TD-1 responses in which T helper cells provide the necessary costimulatory signals and TD-2 responses in which the CD40–CD40L interaction is orchestrated by innate cells (6).

Although fish IgM^+^ B cells fully responded to TI-1 antigens, this stimulation was not achieved with TI-2 antigens that do not provide a costimulatory signal through the presence of microbial products, pointing to stimulation through TLRs or other innate receptors as an essential requirement for B cell activation in teleost. It has become evident in the past years that B lymphocytes are cells particularly qualified to integrate signals from specific antigens through their recombined BCR with innate signals provided by innate receptors such as TLRs (69). In rainbow trout, IgM^+^ B cells were shown to express all TLRs known to date in this species (70). However, in mammals, how different B cell subsets respond to TLR ligands differs considerably as a result of a specific range of expressed TLRs in each cell subset. Thus, for example, while human B cells do not constitutively express TLR4 and consequently are unresponsive to LPS stimulation, LPS stimulates proliferation, cytokine secretion, and CSR in murine B cells that constitutively express TLR4 (71). In rainbow trout, despite the fact that a homolog of TLR4 has not been found to date in salmonids, IgM^+^ B cells have been shown to be extensively activated by LPS (72). In our study, TNP-LPS induced significant downregulation of the transcriptional levels of several genes that were assigned as TLR13, TLR9, and TLR2. Whether one of these TLRs is directly implicated in the recognition of LPS in fish is still unknown. TLR13 has been reported as an endosomal receptor that recognizes bacterial RNA both in mammals and fish (73, 74), while TLR9 is another endosomal receptor activated in response to unmethylated CpG motifs (75); therefore, the mechanism through which the transcription of these receptors is regulated in response to TNP-LPS is not easily inferred. Furthermore, three of the sequences that were assigned as TLR13 were also significantly downregulated in response to TNP-KLH. TLR2, on the other hand, is a surface receptor known to recognize a wide range of bacterial antigens in mammals (76); thus, it may be possible that it is one of the innate receptors responsible for the recognition of LPS by trout IgM^+^ B cells, and this is something that should be further investigated.

Although the general line of thought establishes that B cells receive signal 2 through T cell help in the case of TD antigens or through TLR signaling in the case of TI responses (3), studies performed in humans revealed that TLR stimulation acts as a “third signal” that couples with BCR cross-linking and T cell help allowing naive B cells to reach a degree of activation not achieved in the absence of innate stimuli (77). The fact that CD40L synergized with TI antigens to activate rainbow trout IgM^+^ B cells also points to teleost TLRs being capable of adding up to BCR and CD40-mediated signals to achieve a higher degree of B cell activation. Similarly, CD40L was shown to act as a costimulatory signal for TI responses mounted in response to a polyomavirus infection in mice lacking T cells (78). Thus, rather than having a fixed dichotomy between TD and TI responses, it now seems that B cells become activated at rising degrees through accumulation of different signals, being that received by innate receptors essential in teleost for a complete cellular activation.

In conclusion, we have demonstrated that teleost fish preferentially respond to TI antigens over TD antigens, in correlation with the many similarities found between fish IgM^+^ B cells and mammalian B1 cells (60). Despite this, fish IgM^+^ B cells responded to TD antigens activating the transcription of several genes related to immune processes, many of which lead to the activation of the NF-κB pathway. Some of these genes transcriptionally regulated in response to TNP-KLH were specific and were not affected by TNP-LPS stimulation, such as CD40 or associated TRAF molecules, demonstrating for the first time in teleost the dichotomy between TD/TI responses at a molecular level. However, additional results further suggest that TD and TI pathways are not completely independent in teleost fish as already suggested in mammals. The demonstration that CD40L synergized with TI antigens revealed that B cells accumulate signals to achieve different degrees of activation, integrating stimulatory effects received through the BCR (signal 1), CD40 (signal 2), or TLRs (signal 3). Finally, we have also demonstrated that fish IgM^+^ B cells are activated by CD40L similarly to mammalian cells. These results *a priori* would seem in contradiction with the lower response elicited by TD antigens, but might be indicating that, in fish, CD40L provided by non-classical helper cell types (TD-2 responses) is more effective than responses modulated by T helper cells (TD-1 responses). In line with this hypothesis, having established that fish DCs express CD40L as many mammalian innate cells also implies that the CD40:CD40L stimulation can be achieved by alternative pathways that involve cell types different than T cells, again pointing to new unconventional pathways to activate B cells.

## Data Availability Statement

Data supporting the findings of this work are available within the paper and its Supplementary Information files. The transcriptional data obtained in this work has been deposited in National Center for Biotechnology Information's Gene Expression Omnibus (79) and is accessible through the GEO Series accession number GSE129092 (https://www.ncbi.nlm.nih.gov/geo/query/acc.cgi?acc=GSE129092). All other data are available from the corresponding author upon request.

## Ethics Statement

The animal study was reviewed and approved by Instituto Nacional de Investigación y Tecnología Agraria y Alimentaria.

## Author Contributions

AG performed and analyzed all the experiments involving CD40L with help from IS. AM-M and PD-R performed all experiments with the antigens alone and PP performed all the RNAseq analysis. CT conceived the work and designed the experiments with help from AG and PD-R. CT wrote the main body of the paper with contributions from all other authors.

### Conflict of Interest

The authors declare that the research was conducted in the absence of any commercial or financial relationships that could be construed as a potential conflict of interest.
