# Supplementary material for: Rainbow Trout IgM+ B Cells Preferentially Respond to Thymus-Independent Antigens but Are Activated by CD40L
Source: Front Immunol. 2019 Dec 17;10:2902. doi: 10.3389/fimmu.2019.02902 (PMC6927014; doi:10.3389/fimmu.2019.02902)
Supplement: Supplementary file 3 [file Data_Sheet_1.pdf]

Supplementary Material

Figure S1

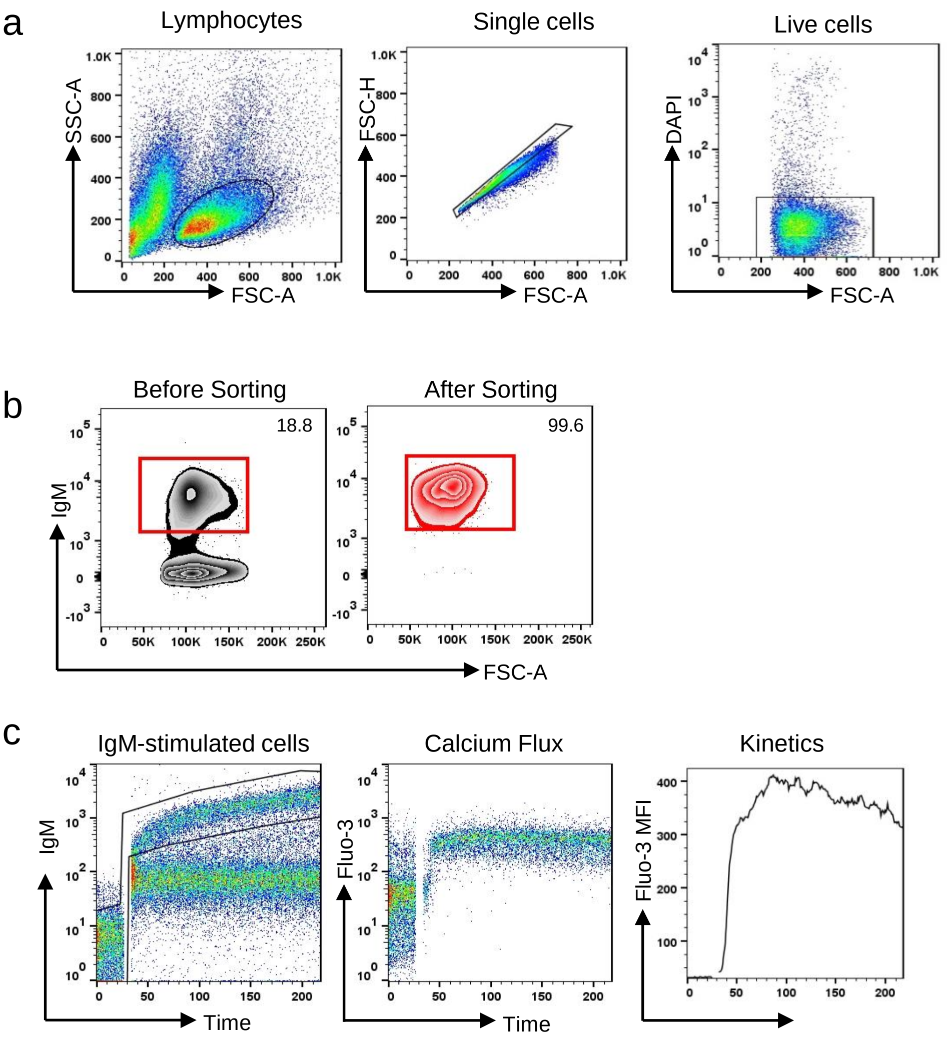

Figure S1. Gating strategy for FACS analysis and isolation of live spleen lymphocytes (a). Flow cytometry analysis of rainbow trout leukocytes isolated from spleen and stained with DAPI. For each sample, FSC/SSC profiles including a defined gate for lymphoid cells are shown (left panel). FSC-H/FSC-A profiles within the lymphoid gate show single cells (middle panel). DAPI negative cells within the singlet population were gated in order to select alive cells (right panel). (b) Dot plot showing IgM^{+} cells before (left panel) and after their sorting (right panel). (c) In the calcium flux assays, after stimulating splenocytes with labelled anti-IgM, anti-IgM-binding cells were gated. Thereafter, the MFI values of Fluo-3 within IgM^{+} B cells were measured. Data are represented as MFI (solid line) of intracellular Ca^{2+} levels in IgM^{+} B cells.

Figure S2

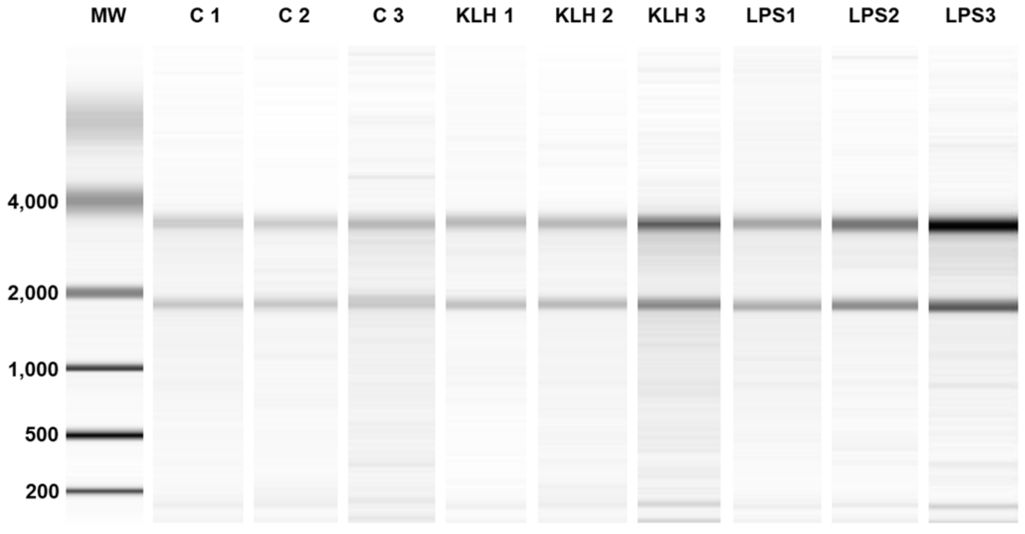

Treatment	Sample	RNA integrity (RIN)	Total number of reads	Total number of Mapped reads	Percentage (%)
	C 1	7.6	16,564,736	9,237,306	55.8
Control	C 2	8	17,335,955	9,228,595	53.2
	C 3	7.5	21,195,727	12,164,154	57.4
	KLH 1	8.5	14,829,738	8,367,432	56.4
KLH	KLH 2	7.9	19,245,594	11,278,701	58.6
	KLH 3	7.3	14,141,353	8,463,178	59.9
	LPS 1	8	12,818,007	8,080,787	63.0
LPS	LPS 2	8.5	16,423,262	9,985,141	60.8
	LPS 3	8.7	17,521,401	11,081,476	63.2

Figure S2. Total RNA quality control and general statistics of Illumina sequencing and mapping. The integrity of total RNA (RNA integrity, RIN) isolated from IgM^+ B cells was evaluated using Bioanalyzer. Lanes: MW, molecular weight; C1, C2 and C3, total RNA from control B cells; KLH1, KLH2 and KLH3, total RNA from TNP-KLH stimulated B cells; LPS1, LPS2 and LPS3, total RNA from TNP-LPS stimulated B cells. The table shows, for each sample, the RNA integrity (RIN), the total number of reads obtained in raw data after quality filtering and the total number of mapped reads against the *Oncorhynchus mykiss* genome, as well as the percentage of mapped reads among the total number of sequences.

Figure S3

a

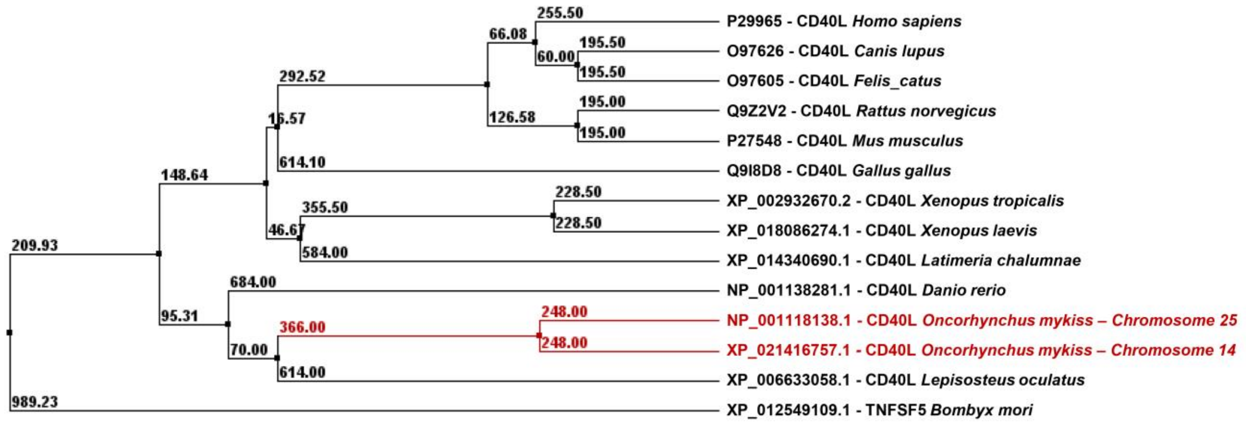

b

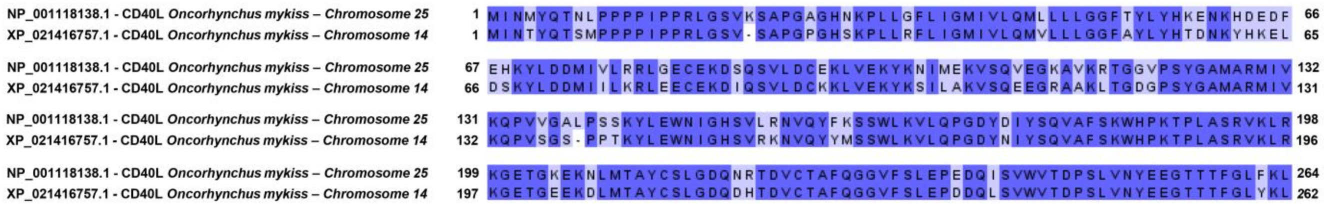

Figure S3. Analysis of the CD40L gene family in *Oncorhynchus mykiss*. (a) Phylogenetic tree showing the average distance constructed from a multiple protein alignment using T-Coffe software implemented in Jalview (v2.10.5). The alignment includes the reviewed amino acids sequences of CD40L from several mammals (*Homo sapiens*, *Mus musculus*, *Rattus norvegicus*, *Canis lupus* and *Felis_catus*) and birds (*Gallus gallus*) available in UniprotKB, together with deduced amino acid sequences of CD40L from amphibians (*Xenopus tropicalis* and *Xenopus laevis*) and fish (*Latimeria chalumnae*, *Lepisosteus oculatus*, *Danio rerio* and *Oncorhynchus mykiss*). The gene TNFSF5 from *Bombyx mori* was used as outgroup. Two gene copies of CD40L was identified in rainbow trout genome in chromosome 25 and 14, respectively. (b) Protein alignment of deduced amino acid sequences from two CD40L *Oncorhynchus mykiss* genes. Dark blue shows conservation in amino acid residue whereas light blue shows differences between the amino acid sequences. A percentage of identity of 81,44% was identified between sequences.

Figure S4

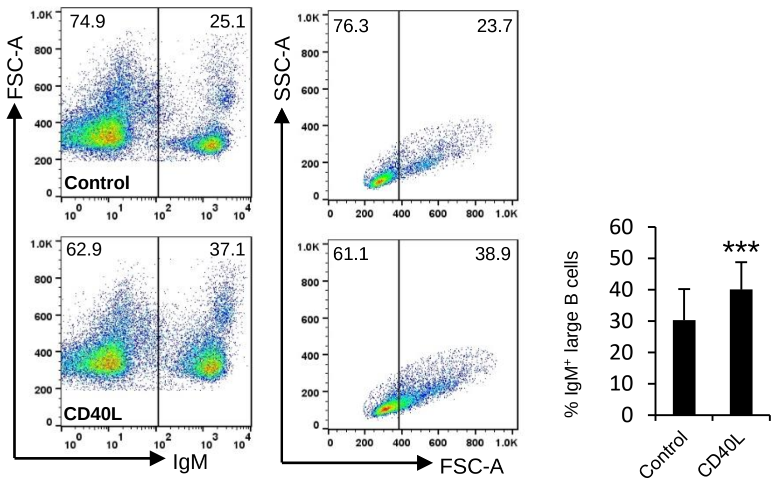

Figure S4. Spleen leukocytes were incubated with CD40L during 72h or left stimulated (control). After this time, cells were labelled with anti-IgM mAb and were analyzed by flow cytometry. Representative dot plots showing the percentage of total IgM^+ cells (left panels) and the percentage of small and large cells within this population (right panels) are shown along with a quantification of the percentage of large IgM^+ B cells in the cultures (n=8).

Figure S5

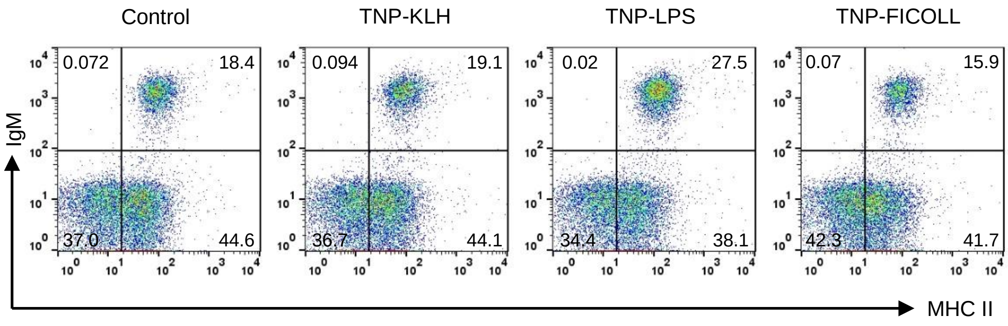

Figure S5. Spleen leukocytes were incubated with TNP-KLH, TNP-LPS, and TNP-Ficoll, or left stimulated (control) during 72 h. After this time, cells were labeled with anti-IgM and anti-MHC II mAbs and the samples analyzed by flow cytometry. Representative dot plots showing MHC II expression on IgM+ B cells from one representative fish are shown.

Table S1: Sequences of primers used in this study

Gene	Forward primer	Reverse primer
EF-1α	GATCCAGAAGGAGGTCACCA	TTACGTTCGACCTTCCATCC
CD40L	GAGTGTGAGAAAGACAGCCAGTCAG	CGTTTGACAGCTTTTCCTTCAACTT
BAFF-R	TGTCTGGATATCAATGGTCGTCATA	CTTTAGCTGGAGGGTTAAGTCTTGC
BCMA	ATGTCAGAAGGACAGTGTGGACTGG	CGGCTCTGGGGCTTTGCTCT
Pax5	ACGGAGATCGGATGTTCCTCTG	GATGCCGCGCTGTAGTAGTAC
Blimp1	AGCTGTCCAACCTCAAGGTCC	TTGCGGCACACCTGGGCATTC
IRF4	CATGGCCACCCTTTCTGACTTCC	GCATACCCCTGCAGCTCAGTGA
IRF8	CCGAGGAGGAGCAGAAGAGTAAAAG	GCGGCATTGAAAGAACCCAT
MHC II	ACACCCTTATCTGCCACGTC	TCTGGGGTGAAGCTCAGACT
CD80/86	GTGTTTCCTGGTTCTGGTATCTA	AACTTGCTGCTCCCTTTCCTC
CD83	GCTGTTGATAGCGGGAGGTA	TGTGGACTCAAGGCAATCTG
